# Characterization of a novel large deletion caused by double-stranded breaks in 6-bp microhomologous sequences of intron 11 and 12 of the *F13A1* gene

**DOI:** 10.1038/hgv.2015.59

**Published:** 2016-02-11

**Authors:** Anne Thomas, Vytautas Ivaškevičius, Christophe Zawadzki, Jenny Goudemand, Arijit Biswas, Johannes Oldenburg

**Affiliations:** 1 Institute of Experimental Haematology and Transfusion Medicine, University Clinic Bonn, Bonn, Germany; 2 Institut d’Hématologie, Hŏpital Cardiologique, Lille, France

## Abstract

Coagulation Factor XIII is a heterotetrameric protransglutaminase which stabilizes preformed fibrin clots by covalent crosslinking them. Inherited homozygous or compound heterozygous deficiency of coagulation Factor XIII (FXIII) is a rare severe bleeding disorder affecting 1 in 2 million individuals. Most of the patients with inherited FXIII deficiency described in the literature carry *F13A1* gene point mutations (missense, nonsense and splice site defects), whereas large deletions (>0.5 kb in size) are underrepresented. In this article we report for the first time the complete characterization of a novel homozygous *F13A1* large deletion covering the entire exon 12 in a young patient with a severe FXIII-deficient phenotype from France. Using primer walking on genomic DNA we have identified the deletion breakpoints in the region between g.6.143,016–g.6.148,901 caused by small 6-bp microhomologies at the 5´ and 3´ breakpoints. Parents of the patient were heterozygous carriers. Identification of this large deletion offers the possibility of prenatal diagnosis for the mother in this family who is heterozygous for this deletion.

## Introduction

Blood coagulation Factor XIII (FXIII) is a protransglutaminase whose main function is to covalently cross link fibrin fibers (via isopeptide-bound glutamyl and lysine residues) and also fibrinolytic inhibitors like α-2-antiplasmin into the fibrin clot to mechanically and chemically strengthen it against fibrinolysis.^1,2^ Zymogenic FXIII circulates in the plasma as a heterotetramer composed of two catalytic A—FXIII-A2—and two carrier B-subunits—FXIII-B_2_.^3^ Calcium binding to the plasmatic FXIII and thrombin cleavage of the N-terminal activation peptide of the FXIII-A subunit leads to the dissociation of FXIII-B_2_ Subunit resulting in the exposure of the catalytic triad to the FXIII substrates.^4,5^

The *F13A1* gene is located on the short arm of Chromosome 6p25–24 and contains 15 exons encoding a protein of 731 amino acids and 83.2 kDa. *F13B* is encoded on the long arm of Chromosome 1q32–32.1 and contains 12 exons encoding for a protein of 641 amino acids reaching 79.7 kDa.^6–9^ Genetic defects in *F13A1* and *F13B* genes result in congenital FXIII deficiency.^10,11^

The most common primary manifestation of severe homozygous deficiency is umbilical cord bleeding after birth followed by severe bleeding diathesis.^12,13^ A spectrum of phenotypes including intracranial bleeding, muscle hematoma, haemarthrosis, abnormal wound healing, menorrhagia and spontaneous abortion have been observed in severely affected patients.^14,15^ Severe form of the disease is rare, affecting one in two million.^13^ Heterozygous deficiencies are caused by heterozygous genetic defects in only one allele of either *F13A1* or *F13B* genes with a mild to asymptomatic phenotype.

A total of 112 different mutations have been reported so far from patients with FXIII deficiency, of which the majority (96) are localized in the *F13A1* gene and only 16 mutations have been detected in the *F13B* gene. A large percentage of the mutations are of the missense type followed by nonsense mutations. Mutations affecting splice sites or resulting in small deletions are less frequent in the *F13A1* gene (*n*=20).^15,16^ Remarkably, only three mutations are large deletions i.e., the deletion reported by Anwar *et al.*, 1998^17^ encompassing exons 4–11, the exon 14 deletion described by Ivaskevicius *et al.*,^18^ and the 3´-untranslated region and a large deletion of the exon 5 found by Otaki *et al.*^19^ The *F13A1* large deletions are either homozygous or have been reported in compound heterozygous form.^17,20,21^ Only a few cases have been characterized with regard to their breakpoints. In this report we characterize the breakpoints of a novel large deletion spanning exon 12 of *F13A1* gene causing a severe FXIII-deficient phenotype in the index patient and a mild and asymptomatic FXIII deficiency in the parents. Small 6-bp microhomologies at the 5´ and 3´ breakpoints are most likely the primary cause for this deletion.

## Materials and methods

### Proband

Blood from a young male of Portuguese origin with severe FXIII deficiency (plasma FXIII activity levels<3%) was genetically analyzed for defects in the *F13A1* and *F13B* gene. The proband showed prolonged and delayed bleeding from the umbilical stump after birth which was successfully treated with plasma-derived FXIII concentrates. The parents of the proband showed mildly decreased FXIII activity levels (51–60% of normal), both parents were clinically asymptomatic. No consanguinity for the parents was reported.

### Isolation of genomic DNA and sequencing

The extraction of genomic DNA (gDNA) from peripheral blood of the proband and his parents followed standard protocols. PCR amplification with *F13A1* and *F13B* gene primers was done for all exons of both genes. The ABI PRISM 3130XL sequencer with the GeneScan 500 LIZ size standard (Applied Biosystems, Darmstadt, Germany) and the Gene Mapper Software 5.0 (Applied Biosystems) was used for sequencing of the PCR products.

### Detection of the breakage points of deletion

The absence of sufficient amount of sample for *in vitro* messenger RNA (mRNA)/complementary DNA transcript analysis prompted us to adopt a primer-walking approach on gDNA for the identification of the deletion breakpoints. Primers started from the 5´-end of exon 11 and 3´-end of exon 13. Intron/exon amplification of the *F13A1* gene was done with a normal Touch-Down (annealing temperature starting with 63 °C, each step reduction of 3 °C to reach 57 °C) PCR with 35 cycles in a total volume of 30 μl containing 50 ng DNA using the Dream-Taq polymerase (Thermo Scientific, Schwerte, Germany). Successively different forward and reverse primers were prepared with the free software tool Primer3 (http://primer3.ut.ee/).^22–24^ The last round of amplification to detect the breakpoints was performed using an iProof High-Fidelity DNA Taq polymerase (Bio-rad, Munich, Germany). Amplified PCR products were sequenced.

### Bioinformatics analysis

We performed two main types of Bioinformatics analysis:

- Sequence analysis. To investigate the potential mechanism causing the deletion, the deleted region was analyzed for different repetitive elements (copy-number variations), like long interspersed nuclear elements, short interspersed nuclear elements and long terminal repeats, known to be associated with deletion-prone regions. Three hundred base pairs up and downstream from the deletion breakpoints were analyzed on the UCSC web server (https://genome.ucsc.edu/). In addition, the Repeat Masker (http://www.repeatmasker.org/) was used to search for repetitive elements. The sequences around the breakpoints were aligned with each other in order to detect identical regions (microhomologies) which can result in deletion during the repair process of a DNA double-strand break.^25–27^
- Splice site prediction. The resulting sequence post deletion was analyzed for new putative splice sites on the splice site detection server (http://wangcomputing.com/assp/).^28^ Only putative splice sites with confidence levels of 0.950 and a score>5.700 for cryptic acceptor site were chosen.

## Results

### Identification of the deletion and its breakpoints

**Figure 1:**
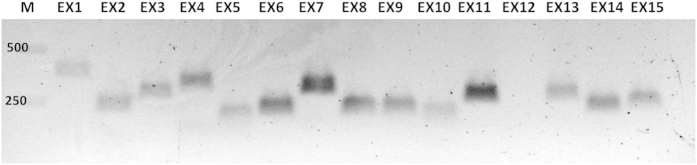
The *F13A1* exon amplification (patient) for sequencing. This figure illustrates an 0.8% agarose gel run for sequencing PCR Product of each of the *F13A1* exons performed with the patient gDNA. Because each amplicon corresponds to the amplification of one exon, it is obvious that exon 12 for the patient was not amplified during these set of sequencing PCR reactions. Lane M: 1  kb ladder, lanes EX1–15: exons 1 to 15.

**Figure 2:**
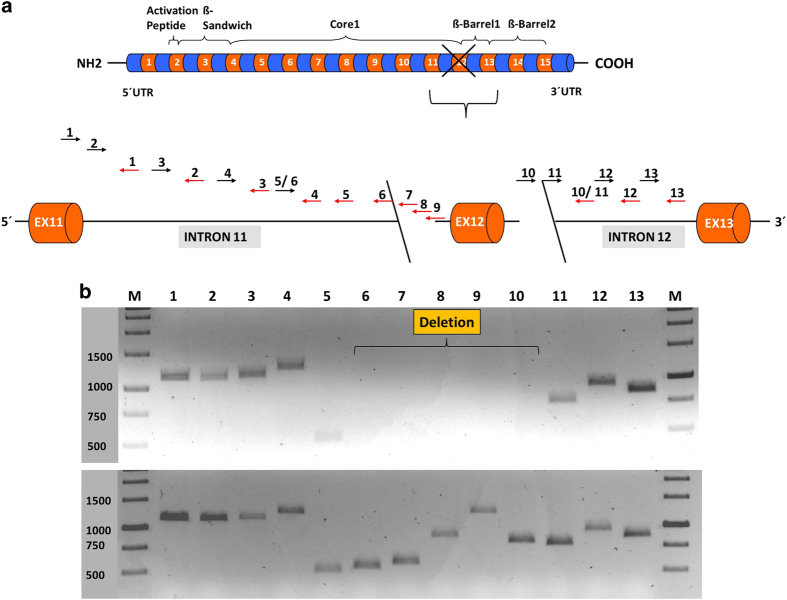
Detection of breakpoint using primer-walking strategy on gDNA of patient. (**a**) Primer walking on gDNA strategy. The upper part of the figure illustrates the general structure of the *F13A1* gene which comprises of 15 exons (orange) and 14 introns (blue). The FXIII-A subunit domains corresponding to the gene/exon–intron length descriptions are also described. The region lost (complete exon 12 and parts of intron 11 and intron 12) as a result of the reported deletion is marked by a cross and brackets. The lower part of the figure shows the region (exon 11 to exon 13) around which the gDNA-walking strategy was designed and performed. The number and series of forward and reverse primers used have been depicted with arrows. The deletion breakpoints which are located in the intronic region of introns 11 and 12 are depicted by slanted lines. Black arrows: forward primers, red arrows: reverse primers, 1 to 13: the complete primer pairs (sequences corresponding to each primer can be found in Supplementary Table 1). (**b**) gDNA-walking PCR analysis of the patient and his parents. This figure is split into two panels. The upper panel shows a 0.8% PCR agarose gel run for amplicons generated from a series of PCR reactions performed using gDNA-walking primers numbered 1 to 13 (sequences have been described in Supplementary Table 1) performed for the patient. The lower panel illustrates the same set of reactions but performed for the gDNA of the parents. The upper panel shows no amplification for the patient receiving in the set of reactions corresponding to lanes 6 to 10 indicating that the deletion breakpoints can be narrowed down to the region corresponding to primer pairs used for amplicons in lanes 6 and 10.

**Figure 3:**
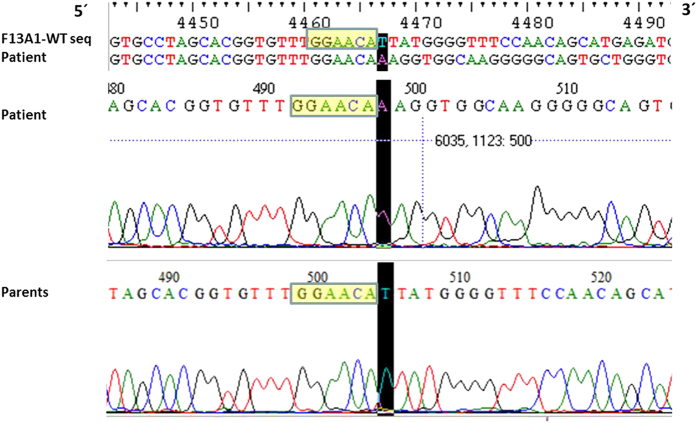
Alignment of the patient sequence with the reported sequence of *F13A1* gene intron 11. This figure illustrates a snapshot of the multiple sequence alignment between sequence reads of intron 11 sequence annotated in Pubmed (top most lane) with multiple sequence reads from the patient for the same region. The black-shaded region highlights in the 5´ deletion breakpoint in the intron 11 region. This region was sequenced using the number 5F (forward) and number 10R (reverse) sequencing primers. Below two snapshots of the electropherogram for the patient as well as for the parents (mother) for the same region are shown. The yellow box marks the 6-bp microhomology region before the 5´ deletion breakpoint.

PCR analysis of the *F13A1* gene revealed that exon 12 could not be amplified in the patient (Figure 1). Analysis of the g.DNA using primer-walking detected a deletion of size 5,885 bp spanning introns 11 and 12 resulting in complete deletion of exon 12 (Figure 2). The breakpoints causing the intronic deletion were narrowed down to the intronic regions between g. 6.143,016–g. 6.148,901. (accession ID: hg19_refGene_NM_000129; https://genome.ucsc.edu/cgi-bin/hgc?hgsid=470422809_v3PeJph508RYF91RcAx8jTmDWvMi&g=htcGetDna2&table=&i=mixed&l=6144084&r=6320834&getDnaPos=chr6%3A6%2C144%2C085-6%2C320%2C834&db=hg38&hgSeq.cdsExon=1&hgSeq.padding5=0&hgSeq.padding3=0&hgSeq.casing=upper&boolshad.hgSeq.maskRepeats=0&hgSeq.repMasking=lower&boolshad.hgSeq.revComp=0&submit=get+DNA). The two primers flanking the deletion were a forward primer 5F (5'- TCCTGGAAAAATACTTGCTC-3') on the 5´-end in intron 11 and a reverse primer 10R ( 5'-TCATTCTACTCACCCATACAAG-3') on the 3´-end of the intron 12 and both were used to define the exact deletion breakpoint (Supplementary Table 1). Using the deletion flanking primers for PCR, both parents were found to be heterozygous carriers for this deletion (Figure 3).

### Analysis for repetitive elements

**Figure 4:**
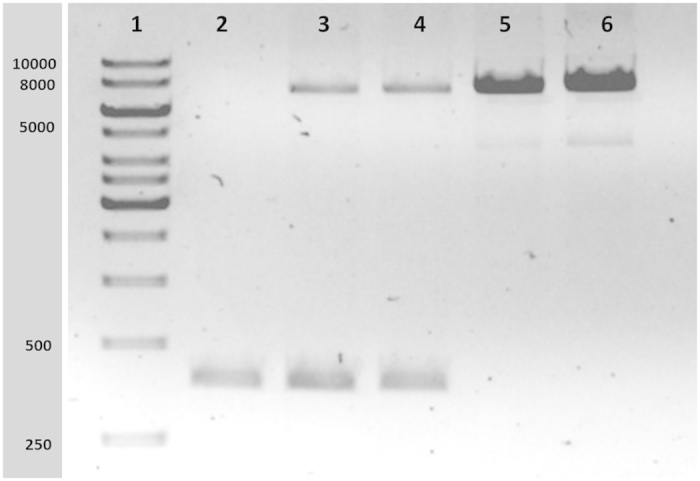
PCR amplification of the region around the breakpoint. This image shows a 0.8% agarose gel run for amplicons amplified using primer numbers 5F (forward) and number 10R (reverse). A Touch-Down PCR (details given in the Materials and methods section) was performed to generate the regions covering the deletion breakpoints. The patient shows only one band (~400 bp) whereas the parents (lane 3 and 4) show two bands (one at ~8 kb and the other at ~400 bp) suggesting that the parents are heterozygous for this particular deletion. The negative control (healthy control DNA samples in lanes 5 and 6) shows the normal product (that is, the higher sized band ~8 kb).

Only one repetitive element, a LINE2 (L2b) repeat fragment covering one of the two deletion breakpoints was detected downstream of the 3´ end. Other repetitive sequences were either located on the deleted fragment itself or found up or downstream from the deletion breakpoints (Supplementary Table 2). Alignment of the 5´- and 3´-end regions bordering the deletion breakpoints showed 6-bp microhomologies between the two regions (Figure 4).

### Splice site prediction

Splice site prediction revealed one putative acceptor site (5'-ttgataacagTGTCTTGATT-3') 1,517 bp upstream of the 5´ deletion breakpoint in intron 11. Splicing with the normal donor splicing site (5'- ttccaagaagGTAATTTACT-3') from exon 11/ intron 11-boundary and this alternative acceptor site leads to the generation of a premature intronic stop codon two amino acids behind the splicing site in intron 11 ( 5'-CAAGAAGTGTCTTGA-3'). This possibility of alternative splicing would either result in nonsense-mediated mRNA decay for the variant allele or result in a truncated protein of 489 amino acids. Splicing using intact splicing sites of exon 11/intron 11-boundary ( 5'-ttccaagaagGTAATTTACT-3') and intron 12/ exon 13-boundary ( 5'-ctcctactagTCAAGAAAGA-3') would result in a truncated protein of 637 amino acids.

## Discussion

Genetic analysis of the index patient resulted in the detection of a novel deletion of 5,885 base pairs spanning introns 11 and 12 lacking exon 12 leading to *FXIII-A* deficiency. The starting point of the deletion is located in the middle of the intronic region from intron 11 and does not destroy the 5´ donor splicing site of exon 11/intron 11 boundaries. The splicing acceptor site at the intron 11/exon 12 boundaries and the 5´ donor splicing site from exon 12/intron 12 are affected by the deletion. The splicing acceptor site of intron 12/exon 13 is intact. This combination generated two possibilities. One of them is that the intact splicing sites are used, resulting in an alternatively spliced mRNA which skips exon 12. An altered-truncated protein, missing parts of the vital catalytic core, and barrel-1 domain would be generated provided the variant mRNA is translated. The other possibility is that the deletion creates an altered reading frame because of a putative acceptor site, which we observe from splice site prediction on remaining contiguous sequence. This gives rise to a severely truncated protein (of 489 amino acids) resulting from a premature stop codon caused by the alternative splicing. In case of a premature stop codon being introduced, the mRNA may be subjected to nonsense-mediated decay, which degrades the variant mRNA before it can be translated.

**Figure 5:**
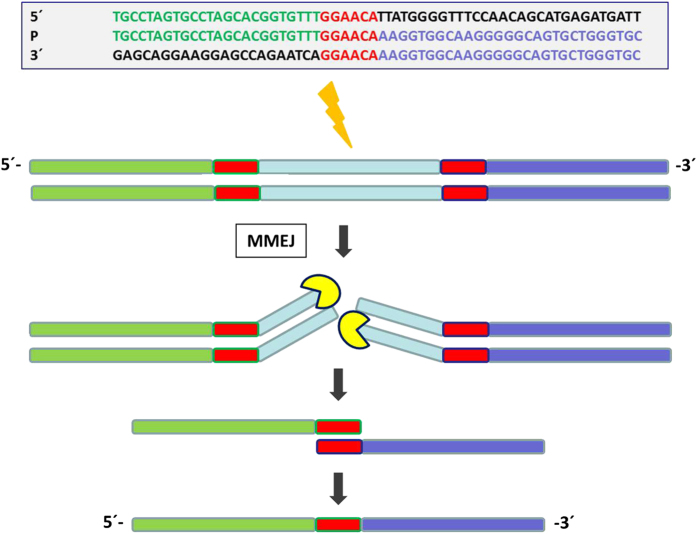
Visualizing the 6-bp microhomologies covering the deletion break points. This image show the double-stranded breaks at the 5´ (green color) and 3´ (blue color) ends surrounded by small 6-bp microhomologies (red). The deletion results from the error-prone microhomology-mediated end-joining pathway repair mechanism which follows this double-stranded break and joins the broken ends.

In the literature there are two recently published papers,^29,30^ which report the association of 6-bp long microhomology with deletion breakpoints in the *F8* and *F9* genes similar to what we detect in our deletion breakpoints. Microhomologies are short homologous regions of DNA and appear with a higher prevalence in potential chromosomal breakpoints of physiological and pathological significance.^25,27,30^ Double-strand breaks in the DNA seem to appear with a higher prevalence in regions bearing such microhomologies.^31,32^ Repair of such DSB is mediated by two main pathways: (i) the non-homologous end-joining pathway and (ii) the homologous recombination pathway.^33^ Recent studies into the repair mechanisms showed the appearance of small deletions or insertions at breakpoint junctions during double-strand break repair mediated by the non-homologous end-joining pathway.^27,34,35^ Our deletion shows neither long homologous regions on the respective 5´ and 3´ ends of the deletion nor is it susceptible to non-homologous recombination, as non-homologous end-joining directly ligates the broken DNA strand ends without filling additional bases on the overhang and also ligates microhomologies <6 bp.^36^ It is also known that other mechanisms like microhomology-mediated repair mechanisms, like microhomology-mediated end-joining pathway, FoSTeS, MMBIR, SRS or BISRS, are responsible for the occurrence of deletions.^31^ The deletion is probably a result of the alternative repair mechanism known as microhomology-mediated end-joining pathway (Figure 5) which has been recently identified.^37^ This alternative mechanism relies on 5–25-bp microhomolgies^35,38^ to perform DBS repair by removing mismatches of overhanging DNA. Therefore we hypothesized that the 6-bp microhomologies observed on either ends of the breakpoints might be the primary cause for this large deletion. Our data is in concordance with the findings of other groups characterizing deletion breakpoints in other genes like coagulation *F8*, *F9* and also the *FOXL2* gene.^28,29,31^

Screening of >100 unrelated patients of Caucasian origin from Bonn region with reduced FXIII levels (residual FXIII activity<70% of normal) by PCR did not detect any carriers for the recently identified mutation. These patients underwent prior analysis by direct sequencing, showing no other *F13A1* and *F13B* point mutations or small deletions/insertions (data not shown). The absence of large deletion involving exon 12 in this cohort indicates that the deletion in homozygous form might have resulted from consanguinity or near consanguinity in the family from France although none was reported in the patient anamnesis. Unfortunately, we had no possibility to screen for this deletion in the populations from Portugal or France.

Unlike homozygous *F13A1* and *F13B* large deletions, heterozygous large deletions can be missed during direct sequencing if primers are located within the deleted fragment. In such conditions techniques such as multiple ligation-dependent probe amplification or the more recently published AccuCopy technique can be used to detect heterozygous deletions.^39^ Unfortunately, the MLPA technique has not yet been developed for the *F13A1* and *F13B* genes complicating detection of heterozygous large deletions in patients with suspected mild (heterozygous) FXIII deficiency.

## Notes

Supplementary Figures/Tables
