# Supplementary material for: Characterization of a novel large deletion caused by double-stranded breaks in 6-bp microhomologous sequences of intron 11 and 12 of the F13A1 gene
Source: Hum Genome Var. 2016 Feb 11;3:15059–. doi: 10.1038/hgv.2015.59 (PMC4760118; doi:10.1038/hgv.2015.59)

**Table S1.** List of the primer pairs and sequences used for amplifying the sequence up and downstream of the deletion break points.


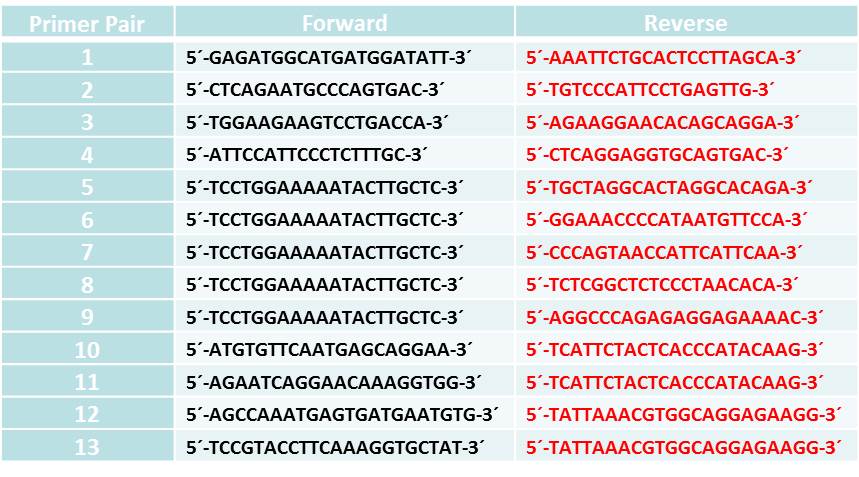


**Table S2.** Summary of the repititive elements detected on analysis of the regions surrounding the deletion break points.


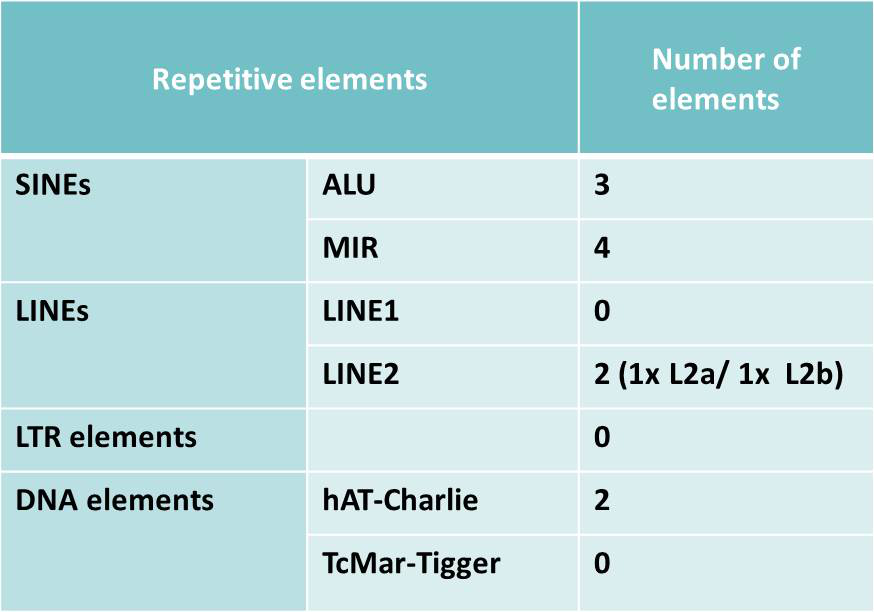

Supplement: Supplementary Figures/Tables [file hgv201559-s1.doc]
